# Assessing Organizational Readiness to Change through a Framework Applied to Hospitals

**DOI:** 10.1007/s11115-022-00628-7

**Published:** 2022-04-06

**Authors:** Irene Gabutti, Christian Colizzi, Tommaso Sanna

**Affiliations:** 1 Department of Biomedical and Neuromotor Sciences, Alma Mater Studiorum - Università Di Bologna, Via San Giacomo 12, 40126 Bologna, Italy; 2 Fondazione Policlinico Gemelli IRCCS, Università Cattolica del Sacro Cuore, Largo Francesco Vito 1, 00168 Rome, Italy

**Keywords:** Readiness to change, Hospital, Framework, Dimensions, Literature review

## Abstract

Understanding and managing hospital Organizational Readiness to Change is a key topic with strong practical implications on society worldwide. This study provides, through a scoping literature review, a framework aimed at creating a road map for hospital managers who are implementing strategic processes of change. Ideally, the framework should act as a check-list to proactively detect those items that are likely to impede successful change. 146 items were identified and clustered into 9 domains. Finally, although built for the hospital setting, similar research approaches could be highly effective also in other large, public organizations.

## Introduction

Although topics such as organizational readiness to change (ORC), organizational resilience and change management are all widely addressed in organizational studies in the private/industrial sector (Grimolizzi-Jensen, 2018), they are, by now, of the utmost importance for large, public organizations too (Sawitri, 2018). In general, managerial revolutions such as the one of New Public Management (Nunes & Ferreira, 2019) have gradually clarified that many challenges typically faced in the private sector are by now just as relevant in the public one. For example, ORC is a highly relevant aspect of public organizations, frequently required to implement managerial tools borrowed from the private industry so to pursue objectives related to both quality *and* efficiency simultaneously (Veillard et al., 2005). ORC is a construct that describes an organization’s capability of implementing a transformation, whether planned or sudden.

A clear example of its relevance can be found in hospitals. These are (frequently public) large and complex organizations which are required to adapt to rapidly changing environments. Although their trends of change are widely studied (Gabutti & Cicchetti, 2020), there exists high variability in their ability of responding to common challenges. For example, this has become evident with the Covid-19 pandemic which has obliged hospitals to face unprecedent and completely unknown scenarios and to rely nearly exclusively on their managerial asset to adapt quickly to an evolving environment (Gibbons et al., 2021).

Therefore, understanding and managing hospital ORC is a key topic with strong practical implications on society worldwide. Hospital ORC has been studied in the past (Vaishnavi et al., 2019). Nevertheless, studies mostly address specific features of hospital ORC and mostly fail in providing a comprehensive framework able to guide managers in the overall assessment and improvement of ORC. Indeed, taking complex decisions when such a comprehensive framework is not available is risky. It is difficult to foresee the interconnected effects a decision implies. Moreover, there may exist numerous organizational and contextual features that could hinder the implementation and success of such decision. In other terms, strategies may fail due to the high number of barriers that impede a concrete process of organizational change (Sicakyuz & Yuregit, 2020).

In this scenario, it is important to provide a concrete framework to classify (and manage) the various dimensions of hospital ORC. This framework is aimed at providing a road map to hospital managers who are implementing strategic processes of change. Ideally, the framework should act as a check-list to proactively detect those items that are likely to impede successful change.

## Background

Healthcare organizations worldwide are undergoing deep transformations to respond to multiple challenges (Daniel et al., 2013). Terms such as "patient-centred care," "clinical pathways," "integrated care” (Daniel et al., 2013; Gabutti & Cicchetti, 2020) are increasingly used in the daily lexicon of those who manage health organizations (Rathert et al., 2013). This means that health care organizations, and hospitals in particular, are facing deep organizational innovations with, for example, transitions from vertical to horizontal organizational models and from managerial approaches based on individual (at the unit level) accountability to assets based on joint accountability (Carini et al., 2020).

In this evolving scenario, coercive isomorphism (DiMaggio & Powell, 1983) is frequently at the basis of hospital compliance with the provisions of national or supranational institutions. Hospitals are obliged to change so to adapt to compulsory indications coming from outside. However, hospitals can also play a proactive role in implementing organizational change (Ribera et al., 2016), giving rise to forms of so-called mimetic isomorphism (Mascia et al., 2014). In this case, they freely choose to implement change and imitate successful strategies observed in similar organizations. Whatever the nature and motives behind organizational change, this must be supported by an adequate contextual and managerial scenario if doomed to succeed.

Implementing organizational change is an unquestionably challenging process due to the many factors that may hinder it. Managers should be fully aware of the organizational dimensions that may affect any transformation process. It is essential to know how to evaluate ORC so to avoid "decoupling phenomena," which imply a discrepancy between theoretical strategic decisions and concrete operational change (Mascia et al., 2014). ORC is indeed considered a critical foundation to implement complex change in healthcare settings successfully (Weiner, 2009). It has been reported that failure to establish adequate readiness accounts for one-half of all ineffective, large-scale organizational change efforts (Weiner, 2009).

Several authors have faced this issue and detected some items which may affect ORC in healthcare organizations. However, most of the published literature is focused on specific aspects of ORC, which cannot be directly translated into holistic assessments of this construct. A conceptual framework to understand factors influencing ORC was provided in a landmark study describing four key constructs that constitute ORC: "*Individual psychological, Individual structural, Organizational psychological and Organizational structural* (Holt et al., 2010a)*.* In other words, factors influencing ORG may either be ascribable to a physical person or to the organization as a whole and may either belong to hard (structural) or soft (psychological) dimensions. Nevertheless, though highly relevant, this study does not provide guidance on the concrete functional dimensions that managers may use to effectively drive change in a hospital.

This study categorizes evidence from extant literature so to identify a complete range of domains able to affect ORC in hospitals, specifying for each their main items and providing an exhaustive framework for managers called to implement change through them.

## Methods

To identify the domains and items that can affect ORC in hospitals, we performed a scoping review of the literature published over the last ten years. The Web of Knowledge database was searched with the following search string:

- *TS* = *(readiness) OR TS* = *(willingness) OR TS* = *(inclination) OR TS* = *(eagerness) OR TS* = *(promptness) OR TS* = *(preparedness)*
- *AND TS* = (chang*) OR TS = (reorganization*) OR TS = (transformation*) OR TS = (metamorphos*) OR TS = (restructur*) OR TS = *(remodelling)*
- *AND TS* = (health*) OR TS = (medic*) OR TS = (hospital*)
- *Indexes* = *SCI-EXPANDED, SSCI, A&HCI, CPCI-S, CPCI-SSH, BKCI-S, BKCI-SSH, ESCI, CCR-EXPANDED, IC Timespan* = *2010–2020*
- *Refined by: WEB OF SCIENCE CATEGORIES: (MANAGEMENT OR OPERATIONS RESEARCH MANAGEMENT SCIENCE).*

Three independent researchers analysed the articles retrieved to assess their relevance to the study's purposes. Articles were included in the study if at least two out of three researchers classified them as potentially relevant. The articles included in the study were analysed to identify a set of domains able to affect hospital ORC as well as their specific items. For each relevant article, all the items detected were clustered into the emerging domains.

## Results

**Fig. 1:**
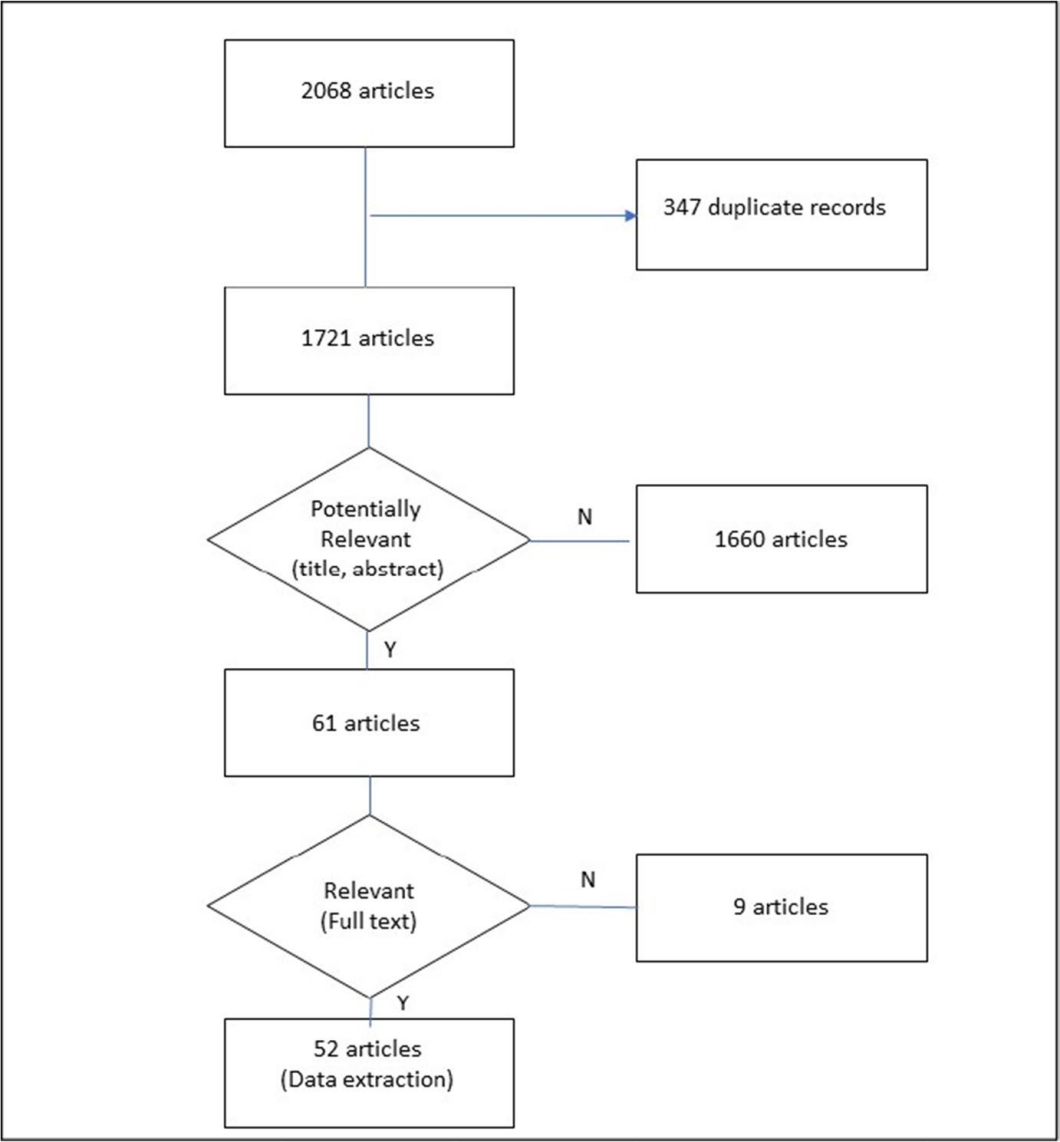
Flow chart of literature review

The search identified 2068 articles. After eliminating 347 duplicate records, three independent researchers performed an analysis of the paper's title and abstract. 61 articles were considered potentially relevant. After an in-depth analysis of full-texts, 52 articles were selected (Fig. 1). 146 items were identified and clustered into 9 domains: Cultural (CULT), Economic and Financial (ECON), External Factors (EXT), Human Resources Management (HRM), Information and Communication Technologies (ICT), Leadership (LEAD), Managerial Accounting (MA), Organizational Structural Factors (ORG). Only one item was not logically attributable to these domains and was therefore included in the domain "Other" (Annex 1). This final domain was then dropped in the analysis due to its scarce relevance and consistency with the overall framework of the study. The individual items were then carefully analysed to assess analogies, with the aim of grouping them into homogeneous super-items. The grouping process was achieved with a Delphi iteration, and the initial 146 items were reduced to 48 super-items, as detailed in Table 1. In this way, for each domain it has been possible to extract the key features and contents that characterize it.

**Table 1:** Domains and super-items

External factors domain	
	Recent trends in healthcare sector
	Resource availiblity in external environment
	Relations with governing bodies
	Relations with other hospitals and providers
	Relations with other stakeholders
Organizational Structural domain	
	Organizational chart
	Responsibility assignement coherence
	Clinical pathways
	Transitional care models
Managerial and accounting tools-Domain	
	Management control systems
	Performance control system
	Presence of a decision support system
	Audits
	Reporting and feedback system
Information and Communication Technology-Domain	
	IC tools in support of clinical data
	IC tools in support of administrative/financial data
	IC tools in support of process/procedure efficiency and logistics
	Inter-organizational communication (other providers)
	Communication with patients
	Communication with governing bodies
	Communication with other stakeholders
	Common program language
Economic and financial resources-domain	
	Budgeting system
	Financial resources
	Technological resources
	Overall sustainability of change over time
Human-resources-management domain	
	Clarity in task demands
	Effectiveness of training programs
	Staffing and workloads
	Organizational and individual conflicts
	Set of skills and competencies of professionals
	Multidisciplinary teams
	Rewards and incentives
	Career coherence
Leadership	
	Staff coaching
	Aligned vision and action
	Staff motivation, committment and engagement
	Staff confidence in task demands
	Collaborative relationships
	Staff participation in decision making
	Staff proactivity and vitaliy
	Perceived managerial support
Organizational-Culture domain	
	Organizational values and beliefs
	Cultural humility and mutual respect
	Enabling and risk-friendly environment
	Patient-centered and final-goal oriented culture
	Internal responsiveness to changing environment
	Climate of trust

The External environmental domain refers to the main trends in the healthcare system (and in the environment in general) in which the hospital operates. New approaches in the provision of care such as patient-centred care, transitional care models and continuity of care to contrast fragmentation, are all examples of super-items within this domain. Furthermore, the set of institutional, normative and reimbursement rules represent other relevant super-items in this area.

The Organizational/structural domain concerns the “hard” dimensions of the hospital. Their organizational charts (whether vertical, horizontal, or matrix-formed) and their overall coherence with their strategic objectives are among the main super-items of this domain. In particular, the presence of organizational units that are adequate in guaranteeing continuity of care and in providing forms of liaison with primary healthcare settings are likely to enable many of the changes hospitals implementing.

The Managerial accounting domain includes super- items concerning the presence of tools aimed at detecting and monitoring relevant indicators which can drive management towards an effective implementation of the organizational strategy. These must be able to support a clear understanding of a hospital’s performance, to be intended in its various acceptations (e.g., clinical, financial, logistic) and both at a macro, meso, and micro level. This will provide a timely access to performance indicators that can support swift decision-making.

The Information and communication technology domain refers to the set of ICT tools which can support a timely and exhaustive access to different types of information, including those on clinical aspects, processes, administrative data. The super-item of a shared (both within the hospital and across different organizations) ICT platform and of a common language in the treatment of data, assumes primary importance in the hospital’s ability of being responsive to change.

The Economic and financial resources domain has to do with both the overall availability of resources as well as with the coherence of their assignment to organizational units (e.g., through budgeting). Such coherence should be interpreted in the light of the hospital’s main objectives.

The domain concerning Human resource management covers the overall set of HRM tools adopted (and properly implemented) in the hospital. Main relevance is assigned to strategic HRM initiatives such as, for example, activity planning and competency modeling. Furthermore, the coherence of career pathways with the main trends of transformation of hospitals is key in the assessment of the sustainability of the latter.

The Leadership domain refers to the general leadership style within the hospital. Although possibly subjective at the individual level, leadership styles can indeed vary across organizations. For example, organizations that encourage shared decision-making and bottom-up communication flows are likely to better respond to timely requests of change that imply an active participation of staff at different levels.

Finally, the Cultural domain concerns the general “atmosphere” felt by staff, with a great difference emerging between organizations that adopt a coercive and corrective approach as opposed to those that appear supportive and encouraging. The extent to which values such as trust, respect, transparency and honesty are pursued, is key in detecting the willingness of staff to implement change.

## Discussion

Organizational readiness to change is a widely explored construct in numerous contexts, including in the healthcare sector. It has been assessed from multiple perspectives, but these are usually limited to one or a few dimensions that may affect it. Comprehensive assessments of organizations’ domains to be managed jointly to implement change effectively, seem to be lacking. This literature review attempts to cover this gap and provides guidance to assess *overall* ORC in hospitals.

This work's pragmatic output provides a basis to build ORC conceptual mapping across different organizational units and areas. For example, some hospitals may be well suited in their HRM asset and lead people towards the intended change successfully. However, they may be anchored to obsolete structural models that slow down the change process. Following this example, if the current organizational chart is not coherent with the new responsibilities professionals are likely to have, the overall result will be disappointing. Again, if an organization is lacking an appropriate managerial accounting system able to monitor the relevant data to implement change, this may not occur even though leadership, for example, is highly effective.

If relevant in general, such an approach appears crucial in the current scenario, greatly affected by the pandemic. Public health organizations may have an interest to assess their overall ORC in order to understand what has hindered or enabled their ability to react quickly to the crisis. Those hospitals that have shown more flexibility and have rapidly adapted to the changing environment have possibly structured a better response to the emergency. ORC is the essence of this intrinsic resilience.

More generally, developing a deep awareness of overall ORC will highly and positively affect organizations' capability of reaching their strategic objectives.

It is worth mentioning a few limits of this study. The main limitation may have to do with the criteria used to cluster items into domains. Given that there is no validated method to do this, researchers have relied on their knowledge of the various domain contents and meanings. Items were grouped accordingly. Nevertheless, whenever consensus was not reached by the first two researchers, the third intervened to mediate conflicts, and a complete consensus was then always reached.

A second limitation has to do with the decision of exploring all available literature in the field without distinguishing by type of hospital (e.g., based on its dimension, mission, location). Although this has been done to detect as much information as possible, there may exist relevant differences between organizations of different types. Future studies should focus on such differences and grasp possible distinctions among their relevant domains and super-items.

## Conclusions

The results of this study provide a starting point to build guiding tools for managers when implementing relevant change within their hospital. Such tools could lead to easy-to-read dashboards, alerting them on the organizational dimensions that are more likely to hinder change in their specific context. This, in turn, would shed light on the problematic aspects they should correct with priority before incurring into unsuccessful, costly plans of change.

At its current stage, the framework provides guidance on the super-items to be assessed but not on the desirable, specific configurations of each. This means that the evaluation of the adequacy of each super-item is left to managers, who must assess them in the light of the specific change process they are willing to implement. Although some “general trends” in the specific configurations of super-items may emerge, these may at times be adequate in some scenarios and not in others. For example, although hospital organizational charts are more and more frequently based on horizontal units of responsibility as a response to the strong need of providing integrated care, a specific hospital may still find it convenient to rely on rather vertical organizational units. Future studies should further decline items, super-items, and domains so to relate them to typical strategies of change.

Finally, although built for the hospital setting, similar research approaches could be highly effective in other large, public organizations. Whether the domains at the basis of ORC in other organizations overlap completely or differ to some extent from those of hospitals, should be further explored.

## Annex 1. Items of hospital organizational readiness to change

**Table:**

Authors	Item	Domain
(Abrahamsen et al., 2017)	Interprofessional collaboration model	CULT
(Amarantou et al., 2018)	Resistance to change is influenced by four main factors (employee-management relationship, personality traits, employee participation in the decision-making process, and job security); disposition towards change, anticipated impact of change and attitude towards change mediate the impact of various personal and behavioral characteristics on RtC	CULT
(Augustsson et al., 2017)	individual- and group-level openness to organizational change	CULT
(Austin et al., 2020)	individual readiness factors, central role of middle manager, how frontline providers and middle managers experienced six readiness factors: discrepancy, appropriateness, valence, efficacy, fairness and trust in management	CULT
(Bastemeijer et al., 2019)	continuous assessment of patient experiences	CULT
(Billsten et al., 2018)	motivational readiness, institutional resources, staff attributes, and organizational climate	CULT
(Castaneda et al., 2012)	community and organizational climate that facilitates change	CULT
(Castaneda et al., 2012)	(1) community and organizational climate that facilitates change, (2) attitudes and current efforts toward prevention, (3) commitment to change, and (4) capacity to implement change	CULT
(Feiring & Lie, 2018)	Three factors were related to capability, including (1) knowledge and acceptability of task shifting rationale; (2) dynamic role boundaries; and (3) technical skills to perform biopsies and aspirations. Five factors were related to motivation, including (4) beliefs about task shifting consequences, such as efficiency, quality and patient satisfaction; (5) beliefs about capabilities, such as technical, communicative and emotional skills; (6) job satisfaction and esteem; (7) organisational culture, such as team optimism; and (8) emotions, such as fear of informal nurse hierarchy and envy. The last two factors were related to opportunity, including (9) project planning and leadership, and voluntariness; and (10) patient preferences	CULT
(Han et al., 2020)	The organization dimension included organizational scale, organizational culture, staff resistance to change, staff training, top management support, and organizational readiness	CULT
(Jakobsen et al., 2016)	Implementing participatory interventions at the workplace may be a cost-effective strategy as they provide additional benefits, e.g., increased social capital and improved organizational readiness for change, that exceed the primary outcome of the intervention	CULT
(Kabukye et al., 2020)	organizational flexibility and collective self-efficacy	CULT
(Karalis & Barbery, 2018)	staff education, and analysing the safety events and sharing the knowledge	CULT
(Kelly, et al., 2017)	relationship between staff perceptions of ORC and the process of innovation adoption: exposure, adoption, implementation and integration into practice	CULT
(Kelly et al., 2017)	organizational functioning, better program resources and specific staff attributes, staff workloads, good organizational climate	CULT
(Mrayyan, 2020)	continuing education courses for staff and focus on teamwork, open communication, total quality management, strategic planning, advanced nursing practice and participatory management	CULT
(Sopow, 2020)	ability to address rapidly evolving external environmental factors	CULT
(Sopow, 2020)	Common understanding of strategy and roadmap, Level of engagement of members and their commitment, Quality and timeliness of decisions, Execution norms that match capabilities to the environment	CULT
(Tummers et al., 2015)	HRM practices are particularly effective for improving proactivity and vitality: high autonomy, high participation in decision making and high teamwork	CULT
(Vaishnavi & Suresh, 2020)	customer-oriented and goal management cultures	CULT
(Vaughn et al., 2019)	poor organisational culture (limited ownership, not collaborative, hierarchical, with disconnected leadership)	CULT
(von Treuer et al., 2018)	capacity to change their organizational climate	CULT
(Willis et al., 2016)	assess cultural change	CULT
(Willis et al., 2016)	existing contextual values and belief	CULT
(Willis et al., 2016)	promoting use of a common program language	CULT
(Willis et al., 2016)	fostering a sense of legitimacy, cultural humility, willingness to engage and mutual respect	CULT
(Alharbi, 2018)	resources are available	ECON
(Bastemeijer et al., 2019)	Organizational barriers:lack of engaged management, no culture of change, lack of financial support. Organizational promoters: organization support system change through engaged leadership; support staff by coaching, provision of information, education, multidisciplinary collaboration	ECON
(Karalis & Barbery, 2018)	Cost was a barrier. Remuneration came in reduction of safety events and costs avoided	ECON
(Kelly et al., 2017)	financial resources	ECON
(Spitzer-Shohat & Chin, 2019)	sustainability of change over time	ECON
(Vaishnavi et al., 2019)	cost effectiveness	ECON
(Alharbi, 2018)	situational factors are aligned	EXT
(Cane et al., 2012)	Environmental Context and Resources', 'Social Influences'	EXT
(Han et al., 2020)	The environment dimension included external pressure, external support, network externality, installed base, and information communication	EXT
(Holt et al., 2010a)	circumstances under which the change is occurring	EXT
(Randall et al., 2020)	organizational context and resources	EXT
(Sopow, 2020)	managers able to identify how internal organizational structures, systems and climates can harmonize with external climates including societal expectations, economic and technological change and public policy	EXT
(Spitzer-Shohat & Chin, 2019)	outer and inner organizational contexts	EXT
(Vaishnavi et al., 2019)	state of affairs, recent trends in healthcare sector	EXT
(Vaishnavi et al., 2019)	environmental scanning, resource availability	EXT
(Vaughn et al., 2019)	dysfunctional external relations with other hospitals, stakeholders, or governing bodies	EXT
(Abrahamsen et al., 2017)	interprofessional collaboration model	HRM
(Al-Hussami et al., 2018)	subjective career success	HRM
(Bastemeijer et al., 2019)	professional barriers: skepticism among staff, difficulty in changing behaviour, level of experience of staff, staff changes at management level	HRM
(Bastemeijer et al., 2019)	Organizational barriers:lack of engaged management, no culture of change, lack of financial support. Organizational promoters: organization support system change through engaged leadership; coaching, information, education, multidisciplinary collaboration	HRM
(Bickerich & Michel, 2016)	executives with high levels of autonomy or high management support benefited from change-coaching	HRM
(Billsten et al., 2018)	motivational readiness, institutional resources, staff attributes, and organizational climate	HRM
(Cane et al., 2012)	'Knowledge', 'Skills', 'Social/Professional Role and Identity', 'Beliefs about Capabilities', 'Optimism', 'Beliefs about Consequences', 'Reinforcement'	HRM
(Feiring & Lie, 2018)	Three factors were related to capability, including (1) knowledge and acceptability of task shifting rationale; (2) dynamic role boundaries; and (3) technical skills to perform biopsies and aspirations. Five factors were related to motivation, including (4) beliefs about task shifting consequences, such as efficiency, quality and patient satisfaction; (5) beliefs about capabilities, such as technical, communicative and emotional skills; (6) job satisfaction and esteem; (7) organisational culture, such as team optimism; and (8) emotions, such as fear of informal nurse hierarchy and envy. The last two factors were related to opportunity, including (9) project planning and leadership, and voluntariness; and (10) patient preferences	HRM
(Han et al., 2020)	The organization dimension included organizational scale, organizational culture, staff resistance to change, staff training, top management support, and organizational readiness	HRM
(Holt et al., 2010a)	psychological factors (i.e., characteristics of those being asked to change)	HRM
(Jackson et al., 2017)	Three related barriers included the need to address: (1) competing organizational demands, (2) differing mechanisms to integrate new interventions into existing workload, and (3) methods for referring patients to disease and self-management support programs	HRM
(Kabukye et al., 2020)	sensitization, training, resolution of organizational conflicts	HRM
(Kampstra et al., 2018)	improving teamwork, implementation of clinical guidelines, implementation of physician alerts and development of a decision support system	HRM
(Karalis & Barbery, 2018)	staff education, and analysing the safety events and sharing the knowledge	HRM
(Karalis & Barbery, 2018)	staff education, and analysing the safety events and sharing the knowledge	HRM
(Kelly et al., 2017)	organizational functioning, better program resources and specific staff attributes, staff workloads, good organizational climate	HRM
(Lim et al., 2019)	Motivational interviewing (MI) is internationally recognised as an effective intervention to facilitate health-related behaviour change. clinical educators could potentially play a central role as change agents within and across the complex clinical system	HRM
(Magdzinski et al., 2018)	preparation strategies such as educational resources, managerial support and personal initiatives	HRM
(Miake-Lye, et al., 2020)	characteristics of individuals	HRM
(Mrayyan, 2020)	To prepare for change, nurse leaders should initiate interventions to enhance organizational readiness and facilitate the integration of change, such as continuing education courses for staff and focus on teamwork, open communication, total quality management, strategic planning, advanced nursing practice and participatory management, especially shared decision-making and policy development	HRM
(Proctor et al., 2019)	implementation climate, participants reported the greatest increases in educational support and recognition for using EBP (evidence-based practices)	HRM
(Randall et al., 2020)	workforce issues	HRM
(Vaughn et al., 2019)	inadequate infrastructure (limited quality improvement, staffing, information technology or resources)	HRM
(Han et al., 2020)	technology dimension included relative advantage, complexity, compatibility, trialability, observability, switching cost, standards uncertainty, and shared business process attributes	ICT
(Kabukye et al., 2020)	Perceived benefits of an electronic health record (EHR) included improved quality, security and accessibility of clinical data, improved care coordination, reduction of errors, and time and cost saving, computer infrastructure, computer skills of staff	ICT
(Kampstra et al., 2018)	high quality database	ICT
(Pfortmiller et al., 2011)	using organizational change management techniques to facilitate adoption of a new clinical information system and discussed development of a change readiness survey tool	ICT
Sopow, 2020	Social technologies ensure educational, support, in- terpersonal communications and other relationships that support care teams and the work of clinical and other staff and effective relationship with patients	ICT
Sopow, 2020	Clinical/work technologies target the use of proper diagnosis and treatment methods technologies, appli- cation of agreed upon standards of care, engaging pa- tients in their treatment, and ensuring effective work process in support of effective care	ICT
Sopow, 2020	Information technologies provide information entry, organization, access, exchange, and reporting activities for effective service and organizational support	ICT
(Vaishnavi et al., 2019)	technology advancement and interdependence among departments	ICT
(Vaishnavi et al., 2019)	innovativeness	ICT
(Vaughn et al., 2019)	inadequate infrastructure (limited quality improvement, staffing, information technology or resources)	ICT
(Willis et al., 2016)	promoting use of a common program language	ICT
(Proctor et al., 2019)	implementation leadership skills to adopt or improve the delivery of EBP (evidence-based practices)	LEAD
(Alharbi, 2018)	organizational members' willingness to accept and implement change	LEAD
(Al-Hussami et al., 2018)	organizational commitment, organizational support	LEAD
(Al-Hussami et al., 2018)	leadership behavior	LEAD
(Al-Hussami et al., 2017)	leadership guidance program that can promote nurses managers' knowledge of leadership and, at the same time, to enhance their leadership competencies and quality of work to promote their readiness for change in healthcare organizations	LEAD
(Amarantou et al., 2018)	Resistance to change (RtC) is (indirectly) influenced by four main factors (employee-management relationship, personality traits, employee participation in the decision-making process and job security); disposition towards change (DtC), anticipated impact of change (AIC) and attitude towards change (AtC) mediate the impact of various personal and behavioral characteristics on RtC	LEAD
(Augustsson et al., 2017)	individual- and group-level openness to organizational change are important predictors of successful outcomes	LEAD
(Austin et al., 2020)	individual readiness factors, and by highlighting the central role of middle manager readiness for change, how frontline providers and middle managers experienced six readiness factors: discrepancy, appropriateness, valence, efficacy, fairness and trust in management	LEAD
(Bakari et al. 2020)	important implication for leaders of organizational change in Pakistan is that they may use this construct to unearth employee level of understanding and attitude towards change initiative to envisage mechanisms to foster employee support for change	LEAD
(Bastemeijer et al., 2019)	professional barriers: skepticism among staff, difficulty in changing behaviour, level of experience of staff, staff changes at management level	LEAD
(Bastemeijer et al., 2019)	Organizational barriers: lack of engaged management, no culture of change, lack of financial support. Organizational promoters: organization support system change throgh engaged leadership; support staff by coaching, provision of information, education, multidisciplinary collaboration	LEAD
(Bickerich & Michel, 2016)	executives with high levels of autonomy or high management support benefited from change-coaching	LEAD
(Billsten et al., 2018)	motivational readiness, institutional resources, staff attributes, and organizational climate	LEAD
(Cane et al., 2012)	'Knowledge', 'Skills', 'Social/Professional Role and Identity', 'Beliefs about Capabilities', 'Optimism', 'Beliefs about Consequences', 'Reinforcement', 'Intentions', 'Goals', 'Memory, Attention and Decision Processes', 'Emotions', and 'Behavioural Regulation'	LEAD
(Castaneda et al., 2012)	attitudes and current efforts toward prevention, commitment to change, and capacity to implement change	LEAD
(Feiring & Lie, 2018)	Three factors were related to capability, including (1) knowledge and acceptability of task shifting rationale; (2) dynamic role boundaries; and (3) technical skills to perform biopsies and aspirations. Five factors were related to motivation, including (4) beliefs about task shifting consequences, such as efficiency, quality and patient satisfaction; (5) beliefs about capabilities, such as technical, communicative and emotional skills; (6) job satisfaction and esteem; (7) organisational culture, such as team optimism; and (8) emotions, such as fear of informal nurse hierarchy and envy. The last two factors were related to opportunity, including (9) project planning and leadership, and voluntariness; and (10) patient preferences	LEAD
(Han et al., 2020)	The organization dimension included organizational scale, organizational culture, staff resistance to change, staff training, top management support, and organizational readiness	LEAD
(Hauck et al., 2013)	Leadership facilitated infrastructure development in three major areas: incorporating evidence-based practice outcomes in the strategic plan; supporting mentors; and advocating for resources for education and outcome dissemination. Transformational nursing leadership drives organizational change and provides vision, human and financial resources and time that empowers nurses to include evidence in practice	LEAD
(Jackson et al., 2017)	Facilitators: significant commitment from the core implementation team and a desire to improve patient outcomes	LEAD
(Jakobsen et al., 2016)	Participatory organizational interventions may improve social capital within teams and between teams and distant leaders and organizational readiness for change	LEAD
(Kabukye et al., 2020)	strategic implementation	LEAD
(Kabukye et al., 2020)	vision clarity, change appropriateness, change efficacy, presence of an effective champion	LEAD
(Kampstra et al., 2018)	engagement and leadership	LEAD
(Karalis & Barbery, 2018)	supportive leadership	LEAD
(Kelly et al., 2017)	Motivation for change	LEAD
(Lim et al., 2019)	Motivational interviewing (MI) is internationally recognised as an effective intervention to facilitate health-related behaviour change. clinical educators could potentially play a central role as change agents within and across the complex clinical system	LEAD
(Lundmark et al., 2020)	line managers' leadership during an organizational intervention. Employee readiness for change was positively related to constructive leadership, and negatively related to both passive and active destructive leadership	LEAD
(Masood & Afsar, 2017)	transformational leadership through psychological empowerment, knowledge sharing, and intrinsic motivation fosters nurse's innovative work behavior	LEAD
(Mazur et al., 2019)	willingness of executive employees to actively support and participate in the change management process	LEAD
(Morin et al., 2016)	relations among latent constructs reflecting change-related beliefs (necessity, legitimacy, support) and psychological reactions (psychological empowerment, affective commitment to change). Our findings suggest that psychological empowerment and affective commitment to change represent largely orthogonal reactions, that psychological empowerment is influenced more by beliefs regarding support, whereas affective commitment to change is shaped more by beliefs concerning necessity and legitimacy	LEAD
(Mrayyan, 2020)	Successful leaders support employees' creative ideas, focus on the timing of the change, and provide training on change management	LEAD
(Nelson-Brantley & Ford, 2017)	attributes of leading change were identified: (a) individual and collective leadership; (b) operational support; (c) fostering relationships; (d) organizational learning; and (e) balance	LEAD
(Nuno-Solinis, 2018)	staff motivation	LEAD
(Nuno-Solinis, 2018)	higher organizational effort	LEAD
(Oygarden & Mikkelsen, 2020)	strategic translations may foster readiness for change	LEAD
(Proctor et al., 2019)	implementation climate, participants reported the greatest increases in educational support and recognition for using EBP (evidence-based practices)	LEAD
(Puchalski Ritchie & Straus, 2019)	higher organizational effort and staff motivation for overcoming barriers and setbacks	LEAD
(Randall et al., 2020)	perceive management to be high quality, they are more supportive of organizational changes that promote evidence-based practice	LEAD
(Schultz et al., 2019)	the failure to manage the people' element and engage employees hampers the success of that change	LEAD
(Sicakyuz & Yuregit, 2020)	staff commitment by including them in decision-making and process changing	LEAD
(Sola et al., 2016)	extra-effort, efficiency and satisfaction	LEAD
(Sola et al., 2016)	questionnaire measures leadership styles, attitudes and behaviour of managers (transformational, transactional and laissez-faire)	LEAD
Sopow 2020	LEADERSHIP: Convergent, generative, unifying	LEAD
Sopow 2020	Common understanding of strategy and roadmap, Level of engagement of members and their commitment to participate, Quality and timeliness of decisions, Execution norms that match capabilities to the environment	LEAD
(Vaishnavi et al., 2019)	organizational leadership	LEAD
(von Treuer et al., 2018)	capacity to change leadership practices	LEAD
(Willis et al., 2016)	foster distributed leadership (informal leaders, including "opinion leaders")	LEAD
(Willis et al., 2016)	align vision and action	LEAD
(Willis et al., 2016)	make incremental changes within a comprehensive transformation strategy	LEAD
(Bastemeijer et al., 2019)	Organizational barriers: lack of engaged management, no culture of change, lack of financial support. Organizational promoters: organization support system change throgh engaged leadership; support staff by coaching, provision of information, education, multidisciplinary collaboration	MA
(Kampstra et al., 2018)	improving teamwork, implementation of clinical guidelines, implementation of physician alerts and development of a decision support system, audits, frequent reporting and feedback, patient involvement, communication, standardization	MA
(Kampstra et al., 2018)	high quality databas	MA
(Pfortmiller et al., 2011)	using organizational change management techniques to facilitate adoption of a new clinical information system and discussed development of a change readiness survey tool	MA
(Sopow 2020)	Administrative technologies address the proper administrative auspices, structures and processes for innovations including design, staffing, training, financial support and evaluation, and coordination with other units-build versus buy, costing, contracting, cost allocation, return on investment are illustrative issue	MA
(Al-Hussami et al., 2018)	organizational commitment, organizational support	ORG
(Bastemeijer et al., 2019)	Organizational barriers:lack of engaged management, no culture of change, lack of financial support. Organizational promoters: organization support system change throgh engaged leadership; support staff by coaching, provision of information, education, multidisciplinary collaboration	ORG
(Benzer et al., 2017)	organization structure dimensions of differentiation and integration impact readiness for change at the individual level of analysis by influencing four key concepts of relevance, legitimacy, perceived need for change, and resource allocation	ORG
(Billsten et al., 2018)	motivational readiness, institutional resources, staff attributes, and organizational climate	ORG
(Han et al., 2020)	The organization dimension included organizational scale, organizational culture, staff resistance to change, staff training, top management support, and organizational readiness	ORG
(Holt et al., 2010b)	level of analysis (i.e., individual and organizational levels)	ORG
(Jackson et al., 2017)	Three related barriers included the need to address: (1) competing organizational demands, (2) differing mechanisms to integrate new interventions into existing workload, and (3) methods for referring patients to disease and self-management support programs	ORG
(Jakobsen et al., 2020)	Participatory organizational interventions may improve social capital within teams and between teams and distant leaders and organizational readiness for change	ORG
(Kabukye et al., 2020)	organizational flexibility and collective self-efficacy	ORG
(Kelly et al., 2017)	organizational functioning, better program resources and specific staff attributes, staff workloads, good organizational climate	ORG
(Minyard et al., 2018)	contextual factors within the organization	ORG
(Mrayyan, 2020)	having developed plans for expanding ambulatory care or enhancing continuity of care, including nurses on all committees, and involving them in policy development and strategic planning efforts	ORG
(Oygarden & Mikkelsen, 2020)	how the use of editing rules in a strategic translation process impacts readiness for chang	ORG
(Randall et al., 2020)	organizational functioning	ORG
(Spitzer-Shohat & Chin, 2019)	outer and inner organizational contexts	ORG
(Tummers et al., 2015)	HRM practices are particularly effective for improving proactivity and vitality: high autonomy, high participation in decision making and high quality teamwork	ORG
(Spitzer-Shohat & Chin, 2019)	process of translating and implementing equity interventions throughout organizations; organizational and patient outcomes	OTHER
